# Vitamin D Deficiency in Leukemia: Implications for Pathophysiology, Treatment, and Supportive Care

**DOI:** 10.3390/nu18142227

**Published:** 2026-07-09

**Authors:** Zahra Eskandari, Ireneusz Ryszkiel, Amirhossein Faghih Ojaroodi, Haniyeh Bazavar, Shayan Keramat, Agata Stanek

**Affiliations:** 1 Department of Hematology, Faculty of Allied Medicine, Bushehr University of Medical Sciences, Bushehr 7514633341, Iran; zhraaeskndrii@gmail.com; 2 Department of Internal Medicine, Metabolic Diseases and Angiology, Faculty of Health Sciences in Katowice, Medical University of Silesia, Ziołowa 45/47, 40-635 Katowice, Poland; ryszkielirek@gmail.com; 3 Student Research Committee, Tabriz University of Medical Sciences, Tabriz 5165665931, Iran; amirfaghih1103@gmail.com; 4 Department of Hematology, Zanjan University of Medical Science, Zanjan 4513956184, Iran; hani79.bazavar@gmail.com; 5 VAS-European Independent Foundation in Angiology/Vascular Medicine, Via GB Grassi 74, 20157 Milan, Italy; shayan.sk1993@gmail.com; 6 Support Association of Patients of Buerger’s Disease, Buerger’s Disease NGO, Mashhad 9183785195, Iran

**Keywords:** vitamin D, leukemia, fat-soluble vitamins, immune–metabolic regulation, hematopoietic homeostasis, vitamin D receptor signaling, supplementation, supportive care, precision nutrition

## Abstract

Leukemia represents a heterogeneous group of hematologic malignancies characterized by the dysregulated proliferation, differentiation, and survival of hematopoietic cells. Vitamin D (VD), a fat-soluble secosteroid hormone, is increasingly recognized not only as a regulator of calcium–phosphate metabolism but also as an important immune–metabolic modulator involved in hematopoietic homeostasis, inflammatory signaling, and cellular fate determination. Growing evidence suggests that VD deficiency may influence leukemogenesis, disease progression, treatment responsiveness, and supportive-care outcomes in patients with leukemia. Mechanistic studies indicate that the active metabolite, 1,25-dihydroxyvitamin D_3_ (calcitriol), modulates leukemic cell biology by activating the vitamin D receptor (VDR) and regulating genomic and non-genomic signaling pathways, including MAPK, PI3K/AKT/mTOR, JAK/STAT, NF-κB, and β-catenin. Through these pathways, VD may affect transcriptional programs governing proliferation, differentiation, apoptosis, autophagy, immune regulation, and treatment resistance. Clinical and observational studies suggest that low 25-hydroxyvitamin D levels may be associated with adverse outcomes in selected leukemia subtypes, including poorer treatment responses, shorter overall survival and relapse-free survival, and shorter time to treatment. However, the available data remain heterogeneous, and causality has not been established. This review provides an integrated translational synthesis of VD deficiency in leukemia, linking molecular mechanisms with subtype-specific clinical evidence and supportive-care implications. Although VD supplementation should not be considered an independent anti-leukemic therapy, individualized assessment and correction of deficiency may represent a rational, low-toxicity component of supportive care. Further prospective and interventional studies are needed to define optimal supplementation strategies, identify responsive patient subgroups, and clarify the clinical relevance of VD status in leukemia.

## 1. Introduction

Leukemia is a heterogeneous group of hematologic malignancies characterized by the uncontrolled proliferation of abnormal white blood cells, which disrupts normal hematopoiesis and immune function [1]. It affects individuals of all ages, although the incidence and subtype distribution vary according to age, genetics, and environmental factors [1]. Advances in diagnostics and treatment have markedly improved outcomes for many patients; however, leukemia remains a leading cause of cancer-related morbidity and mortality worldwide. Globally, leukemia is estimated to have accounted for 66,890 new cases and 23,540 deaths in 2025. The geographic distribution of leukemia is universal, with higher prevalence and overall mortality in more developed countries. However, the mortality rate is higher in developing countries [2]. Understanding the etiopathogenesis, risk factors, and potential metabolic influences for leukemia is essential for developing more effective prevention and therapeutic strategies [1,3].

Vitamin D (VD) is a fat-soluble secosteroid hormone produced in the skin and obtained from dietary sources and supplementation. It plays a vital role in multiple physiological processes, most notably bone health, by regulating calcium and phosphate homeostasis [4]. VD deficiency is defined by plasma levels of 25-hydroxyvitamin D (25(OH)D) below specific thresholds, commonly cited as 25 or 50 nmol/L. The global burden of deficiency is substantial, with regional prevalence estimates ranging from roughly 8 to 18% and 24 to 49% depending on the threshold used and the population studied [4,5]. Although the proportion of individuals with deficiency declined slightly between 2000–2010 and 2011–2022, the condition remains widespread [6].

Beyond its classical functions, VD exerts pleiotropic effects on cell proliferation, differentiation, apoptosis, and immune modulation through the vitamin D receptor (VDR), which is expressed in a wide range of tissues, including hematopoietic cells [7]. Epidemiological and experimental studies have linked adequate VD status with favorable outcomes in several cancers, and there has been growing interest in its potential influence on hematologic malignancies and immune competence [8,9]. The relationship between VD deficiency and leukemia has garnered increasing attention, with observational data suggesting associations between low VD levels and altered immune function, higher infection rates, and potentially worse clinical trajectories in hematologic malignancies [8,10].

However, the findings remain heterogeneous across studies, with inconsistencies in study design, study populations, and VD assessment timing. Clarifying the nature and direction of this relationship is critical to determine whether VD deficiency is a contributing factor to leukemogenesis, a consequence of disease, or a modulator of disease progression and treatment response. The aim of this review is to synthesize current evidence on the relationship between leukemia and VD status and to clarify its potential mechanistic, prognostic, and supportive-care relevance.

The added value of this review lies in its integrated translational perspective. Unlike previous reviews focusing mainly on epidemiological associations or isolated mechanisms, this manuscript combines evidence on VD deficiency in leukemia with mechanistic data on VDR-mediated genomic and non-genomic signaling, including the MAPK, PI3K/AKT/mTOR, NF-κB, JAK–STAT, and β-catenin pathways. We also review subtype-specific evidence across the major leukemia subtypes, with the depth of discussion reflecting the quantity and quality of available data for each entity, thereby linking VD status with disease biology, treatment response, prognosis, and supportive care. Furthermore, we emphasize the concept of individualized VD assessment and supplementation as a potential low-toxicity supportive strategy while clearly distinguishing it from disease-modifying anti-leukemic therapy.

### Literature Search Strategy and Study Selection

This narrative review was based on a structured literature search conducted in PubMed/MEDLINE, Scopus, and ScienceDirect. The search was designed to identify experimental, observational, clinical, and review-level evidence concerning vitamin D status, vitamin D receptor signaling, and leukemia-related outcomes. The following combinations of keywords and Boolean operators were used: “vitamin D” OR “25-hydroxyvitamin D” OR “25(OH)D” OR “calcitriol” OR “vitamin D receptor” OR “VDR” AND “leukemia” OR “acute myeloid leukemia” OR “AML” OR “acute lymphoblastic leukemia” OR “ALL” OR “chronic lymphocytic leukemia” OR “CLL” OR “chronic myeloid leukemia” OR “CML” OR “hematologic malignancy” OR “lymphoma” OR “myelodysplastic syndrome” OR “myeloproliferative neoplasm”. Additional searches combined these terms with “prognosis”, “overall survival”, “relapse-free survival”, “treatment response”, “immune regulation”, “differentiation”, “apoptosis”, “autophagy”, “supportive care”, and “supplementation”.

The search covered articles published from database inception to 7 March 2026. Eligible publications included original clinical studies, observational cohort studies, case–control studies, interventional studies, experimental in vitro and in vivo studies, meta-analyses, and relevant reviews addressing the relationship between VD deficiency, VD signaling, and leukemia pathophysiology, prognosis, treatment response, or supportive-care outcomes. Studies were included if they reported data on VD status, VD metabolism, VDR-related mechanisms, or VD supplementation in leukemia or closely related hematologic malignancies.

The exclusion criteria were articles not available in English; conference abstracts without full-text availability; duplicate publications; studies unrelated to leukemia or hematologic malignancies; articles addressing VD only in non-hematologic cancers without mechanistic relevance to leukemia; and papers without sufficient relevance to VD biology, VDR signaling, clinical outcomes, or supportive care.

## 2. Vitamin D Biology

### 2.1. Metabolism

VD is obtained from sunlight exposure, food, or supplements in an inactive form [11,12]. After intestinal absorption, VD is incorporated into chylomicrons with the assistance of bile acids and transported through the lymphatic system into the circulation. Some VD is stored in adipose tissue and muscle, while the remainder is transported to the liver, where it binds to vitamin D-binding protein (VDBP) for further metabolic processing [13,14,15].

VD3 is formed in the skin when 7-dehydrocholesterol is exposed to UVB radiation, producing previtamin D3, which is then converted into stable VD3. Prolonged sun exposure leads to the formation of inactive byproducts, thereby preventing VD toxicity. Cutaneous VD3 production is influenced by solar angle, skin pigmentation, age, season, latitude, and geographic location [16,17].

**Figure 1:**
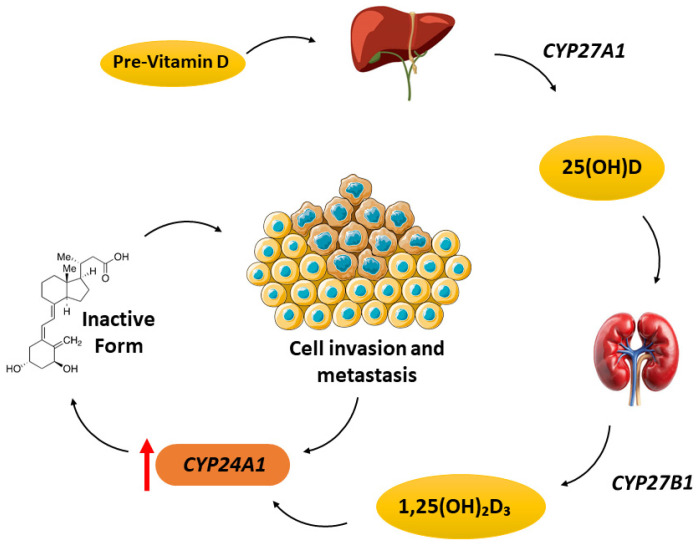
Schematic representation of VD metabolism and its role in cancer cell invasion and metastasis. VD is converted to 25(OH)D in the liver by CYP27A1 and subsequently to the active metabolite 1,25(OH)_2_D_3_ in the kidney by CYP27B1. CYP24A1 catalyzes the degradation of 1,25(OH)_2_D_3_ to inactive metabolites, including calcitroic acid, thereby potentially reducing VD-mediated antitumor activity and promoting tumor progression and metastasis. The red upward arrow (↑) indicates an increase. 25(OH)D: 25-hydroxyvitamin D.

The cytochrome P450 superfamily includes the three primary enzymes involved in VD metabolism: CYP27A1, CYP27B1, and CYP24A1. CYP27A1 converts VD to 25-hydroxyvitamin D [25(OH)D] in the liver. Complete activation occurs primarily in the kidney, where CYP27B1 catalyzes the conversion of 25(OH)D into the active form, 1,25-dihydroxyvitamin D3 [1,25(OH)_2_D_3_], which mediates the major physiological effects of VD in humans [18]. Elevated concentrations of 1,25(OH)_2_D_3_ rapidly stimulate CYP24A1, which subsequently metabolizes 1,25(OH)_2_D_3_ into inactive metabolites, including calcitroic acid. Upregulation of CYP24A1 may reduce local VD activity and has been implicated in cancer progression, particularly because this enzyme is frequently overexpressed in cancer tissues (Figure 1) [18].

CYP24A1 has been widely studied in cancer biology and is frequently overexpressed in several malignancies, including colorectal, lung, breast, and prostate cancers. This overexpression is associated with tumor progression, reduced antitumor activity of 1,25(OH)_2_D_3_, and enhanced cancer cell survival. Experimental studies have also shown that inhibiting CYP24A1 can restore the anticancer effects of VD and suppress tumor growth, highlighting its potential as a therapeutic target [19,20,21,22,23].

In hematologic malignancies, alterations in VD metabolism and signaling may further influence disease behavior. Dysregulated expression of vitamin D-related enzymes, particularly CYP24A1, may reduce the local availability of active VD metabolites and impair VD-mediated biological effects.

Overall, CYP24A1 plays a central role in VD metabolism and may represent a key link between VD signaling and cancer biology, with potential relevance to hematologic malignancies.

### 2.2. Signaling

The biological effects of VD are mediated through its binding to the nuclear vitamin D receptor (VDR), which acts through both genomic and non-genomic pathways.

In the genomic pathway, the VD–VDR complex heterodimerizes with the retinoid X receptor (RXR) and translocates to the nucleus, where it binds to vitamin D response elements (VDREs) and regulates the transcription of genes involved in cell-cycle control, differentiation, and metabolism [15].

In addition to genomic signaling, VD induces rapid non-genomic effects that occur too quickly to be explained by transcriptional regulation alone. These effects involve the activation of intracellular signaling molecules such as PI3K, phospholipase C, phospholipase A2, and p21ras, leading to the generation of second messengers, including calcium, cAMP, and phosphoinositides. This cascade activates downstream kinases such as MAPK, PKC, Src, and Ca^2+^/calmodulin-dependent kinase II, as well as ion channels regulating calcium and chloride fluxes [24,25,26,27].

VDR expression is dynamically regulated in cancer and may vary according to tumor type, tissue context, and VD availability. It is notably high in papillary thyroid carcinoma and breast cancer [28,29]. Similarly, CYP27B1 expression in malignancies may follow a comparable pattern, suggesting that local VD metabolism and VDR signaling may be altered in tumor tissues [30,31]. Higher VDR expression has been associated with improved clinical outcomes in several cancer types, including colorectal cancer, where stromal VDR expression correlates with better survival [32].

**Figure 2:**
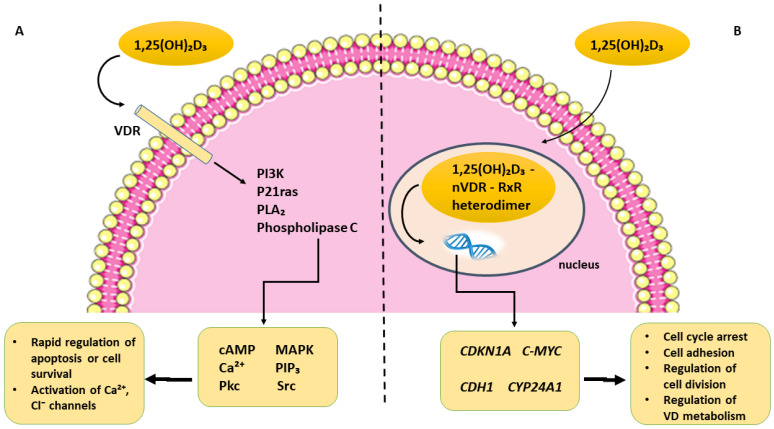
Schematic illustration of the genomic and non-genomic signaling pathways of 1,25(OH)_2_D_3_. (**A**) Non-genomic signaling via membrane-associated VDR activates the PI3K, MAPK, and PLC pathways, leading to rapid regulation of apoptosis and ion channel activity. (**B**) Genomic signaling through the nuclear VDR–RXR heterodimer regulates gene expression associated with cell-cycle control, adhesion, and vitamin D metabolism. MAPK: mitogen-activated protein kinase; PIP3: phosphatidylinositol-3,4,5-trisphosphate; PKC: protein kinase C; cAMP: cyclic adenosine monophosphate; CDKN1: cyclin-dependent kinase inhibitor 1; c-MYC: cellular MYC oncogene; CDH1: cadherin 1, E-cadherin; CYP24A1: cytochrome P450 family 24 subfamily A member 1; VDR: vitamin D receptor; RXR: retinoid X receptor; PLA2: phospholipase A2.

VD also modulates tumor biology through both genomic and non-genomic mechanisms, influencing pathways involved in apoptosis and cell survival. These signaling cascades, including the MAPK and PI3K/AKT pathways, and their detailed roles in leukemogenesis are discussed further in Section 6 [33,34,35] (Figure 2).

Overall, the upregulation of VDR in tumor and stromal compartments highlights the therapeutic potential of VD signaling in cancer, although its role may be context-dependent and complex [36].

## 3. Vitamin D Deficiency: Etiology and Risk Factors

One of the key physiological roles of VD, a lipophilic secosteroid hormone, is the regulation of calcium and phosphate metabolism, which is essential for maintaining bone health [4]. Globally, approximately 8–18% and 24–49% of the population are affected by VD deficiency when defined as plasma 25(OH)D levels below 25 and 50 nmol/L, respectively. Although the prevalence declined slightly between 2000–2010 and 2011–2022, it remains high [2,6,37]. 25(OH)D concentrations are reported in ng/mL or nmol/L, with 1 ng/mL corresponding to 2.5 nmol/L. However, several technical issues should be considered when assessing VD status.

Two main types of assays are used to quantify 25(OH)D: immunoassays, which are commonly used in clinical practice, and chromatography-based methods, which are generally regarded as the reference standard in research settings. Differences in assay methodology between laboratories can result in substantial variability in test results. This variability has contributed to the development of standard reference materials for VD by the National Institute of Standards and Technology in the United States [38].

The total circulating 25(OH)D consists of 25(OH)D_2_ and 25(OH)D_3_; however, not all immunoassays used in clinical practice accurately detect 25(OH)D_2_, which may lead to the underestimation of total 25(OH)D levels. Additional sources of measurement error include interference from other VD metabolites, which may account for approximately 2–20% of total measured 25(OH)D [39]. VD can be obtained from several sources, including food, supplements, and, during certain seasons, sufficient skin exposure to solar ultraviolet radiation, which converts 7-dehydrocholesterol, a cholesterol precursor, through photochemical and thermal processes [40].

Serum VD levels are influenced by several factors, including diet, age, sunlight exposure, season, latitude, skin pigmentation, and lifestyle. VD deficiency is common in infants and young children, particularly between 4 months and 2 years of age, due to rapid skeletal growth [41]. Outdoor sunlight exposure is an important source of VD in children. However, children with darker skin pigmentation require longer UVB exposure to produce equivalent amounts of VD compared with children with lighter skin [42].

Risk factors for VD deficiency include premature birth, dysmature birth, darker skin pigmentation, limited sunlight exposure, obesity, malabsorption, and advanced age, as aged skin produces substantially less VD than younger skin. Historically, rickets was highly prevalent in large cities around 1900. Today, in countries such as the Netherlands, it is more commonly observed among immigrant children, largely due to reduced sunlight exposure, darker skin pigmentation, and dietary factors. The prevalence of VD deficiency is also high among older adults, particularly residents of nursing homes and patients with hip fractures [43,44].

VD deficiency shows substantial geographic variation worldwide, reflecting differences in sunlight exposure, latitude, dietary intake, supplementation practices, skin pigmentation, and lifestyle [37,43]. Higher serum 25(OH)D levels have been reported in Scandinavian countries, whereas lower levels have been observed in Southern Europe. This pattern may reflect differences in supplementation habits, skin pigmentation, sun-exposure behavior, and lifestyle. VD deficiency is particularly common among institutionalized older adults, with prevalence rates exceeding 75% in some nursing-home populations [45]. VD insufficiency is also more frequent in individuals with highly pigmented skin, in whom ultraviolet radiation is less efficient in stimulating cutaneous VD synthesis [45]. A high prevalence of VD deficiency has been reported among non-Western immigrants in the Netherlands [46], and similar findings have been observed in the Middle East, where lifestyle-related factors may contribute substantially [47].

## 4. Vitamin D Status in Leukemia

VD has been shown to influence malignant hematological cells by promoting differentiation in acute myeloid leukemia (AML) and inducing apoptosis in leukemia and lymphoma cell lines [10]. However, the concentrations required for these antitumor effects are often associated with hypercalcemia in vivo [48]. To overcome this limitation, strategies such as the development of VD analogs, combination regimens with chemotherapy, and alternative delivery approaches are being investigated to enhance efficacy while minimizing toxicity [10].

The VD activity in hematological cells depends on the interaction between the VD receptor (VDR) and retinoid X receptor (RXR). In a study of 137 patients with hematologic malignancies and 60 healthy controls, ELISA and RT-PCR analyses demonstrated significant differences in VD levels and VDR/RXR expression, with VD status correlating strongly with disease presence (*p* < 0.001). Age was also identified as an important factor influencing cancer risk and subtype distribution, suggesting that disruption of the VDR–RXR axis may affect DNA damage repair mechanisms [49].

Low circulating 25(OH)D_3_ levels have been associated with increased cancer burden and poorer therapeutic responses in hematologic malignancies. In a cohort of 105 leukemia patients, approximately 72% were classified as VD-deficient or -insufficient, with lower levels correlating with more aggressive disease features [50].

A meta-analysis of seven studies including 2643 patients further confirmed that low 25(OH)D_3_ levels are associated with worse clinical outcomes, including reduced overall survival (OS) and relapse-free survival (RFS) [51]. Similarly, another meta-analysis reported that low-VD status at diagnosis is linked to poorer prognosis across multiple hematologic malignancies and lymphoma subtypes, including DLBCL, FL, HL, and TCL, as well as in hematopoietic stem cell transplantation settings [8].

Most hematologic malignancies express VDR at relatively high levels, suggesting potential responsiveness to VD-mediated effects. In tumors with reduced VDR expression, restoring receptor activity may enhance therapeutic sensitivity [10].

VD signaling may also interact with dysregulated pathways such as JAK–STAT, which is commonly altered in various leukemias and lymphomas, including large granular lymphocytic leukemia, T-cell lymphomas, AML, B-CLL, and T-ALL [52,53,54,55,56,57].

Overall, low-VD status has been consistently associated with poorer prognosis in hematologic malignancies compared with normal VD levels [51]. However, these associations should be interpreted cautiously because most available clinical evidence is observational.

From a system medicine perspective, circulating VD levels are influenced by multiple interrelated determinants, including dietary intake, supplementation, sunlight exposure, geographic location, seasonality, obesity, physical activity, inflammatory status, liver and kidney function, comorbidities, and treatment-related effects. Many of these factors may simultaneously influence both VD status and leukemia outcomes, representing important confounders in observational studies. Therefore, reported associations should be interpreted with caution due to this multifactorial regulation [58].

A summary of the included studies, including VD assay methodologies and the availability of assay standardization data, is provided in Table 1.

Overall, significant heterogeneity exists in VD measurement methods across studies, and standardization using reference methods such as LC-MS/MS or NIST-traceable assays was rarely reported, which may limit cross-study comparability.

## 5. Vitamin D Status in Subtype-Specific Leukemia

It should be noted that the evidence base is unevenly distributed across leukemia subtypes. Acute myeloid leukemia (AML) and chronic lymphocytic leukemia (CLL) are supported by a larger number of clinical and mechanistic studies, whereas evidence for acute lymphoblastic leukemia (ALL) and chronic myeloid leukemia (CML) remains more limited. Accordingly, the depth of discussion in this section reflects the relative availability and strength of the published evidence.

Recent research suggests that serum 25(OH)D_3_ levels may influence outcomes AML. In particular, patients with FLT3-ITD mutations often show lower VD levels, suggesting that VD status may represent a potentially modifiable and cost-effective prognostic factor, although its correction has not yet been proven to improve prognosis [59]. A study of 97 newly diagnosed AML patients undergoing intensive treatment found that 65% were VD-deficient or -insufficient, with 35% and 30% categorized as insufficient and deficient, respectively. Patients with VD levels below the normal range exhibited worse relapse-free survival (RFS) [59]. Additional evidence indicates that circulating 25(OH)D_3_ levels decline further in patients experiencing relapse compared with those in complete remission. Low VD levels have also been linked to poorer overall survival following azacitidine therapy, as well as higher rates of febrile neutropenia and hospitalization [50,60].

Other studies including more than 500 patients with AML, chronic myeloid leukemia, juvenile myelomonocytic leukemia, myelodysplastic syndrome, and primary myelofibrosis showed that patients with lower VD levels had significantly poorer overall survival (OS), although substantial heterogeneity was observed [8,23,24,25]. Progression-free survival (PFS), analyzed in 384 patients from three cohorts, was also significantly poorer among patients with lower VD status [59,61].

Mechanistically, VD3 has been shown to inhibit human myeloid leukemia cells by promoting autophagy and differentiation. It upregulates Beclin-1, a key autophagy mediator; when Beclin-1 is depleted, autophagy is blocked, apoptosis is triggered, and differentiation is impaired, although cell-cycle arrest may remain unaffected [62]. This suggests that autophagy and differentiation can be uncoupled from apoptosis under certain conditions, with autophagy supporting differentiation while suppressing apoptosis [62]. Furthermore, global downregulation of mTOR enhances VD3-driven Beclin-1 expression, increasing autophagic death, whereas combining VD3 with an apoptosis-inducing agent may yield opposite effects. Taken together, Beclin-1 appears to be a pivotal link among autophagy, differentiation, and apoptosis, positioning VD3 as a multifaceted agent that suppresses leukemia cells through both differentiation and autophagy pathways [62].

In a study comparing 54 AML patients with 55 matched controls, investigators measured the expression of VDR and enzymes involved in VD processing, specifically CYP27B1 and CYP24A1, along with related serum proteins. They assessed associations between these molecular markers and clinical outcomes, including fever and neutropenia duration, length of hospital stay, achievement of complete remission, and overall survival [63]. The findings showed that AML patients had significantly higher CYP24A1 expression and higher serum levels of CYP27B1 and CYP24A1 proteins. Notably, CYP24A1-related measures, including gene expression, blood levels, and serum protein levels, were elevated in AML. However, overall VD status and other enzyme levels did not demonstrate a strong or consistent association with treatment outcomes, with the exception of CYP24A1-related markers [63]. In addition, a separate study found that a single-nucleotide polymorphism in the VDR gene, **r**s10783219, correlated with poorer treatment response in AML, including lower complete remission rates, shorter relapse-free survival, and reduced overall survival [59].

Beyond baseline biology, calcitriol analogs with side-chain modifications, such as PRI-1906 and PRI-1907, were observed to promote greater differentiation of AML blast cells than calcitriol alone. CD14 protein levels increased in response to these analogs or calcitriol treatment. Interestingly, the enhanced differentiation induced by these analogs was more pronounced in blasts from patients with a normal or near-normal karyotype, particularly those with NPM1 mutations, whereas cells from patients with FLT3 mutations showed the weakest differentiation response. Overall, these findings point to a nuanced role for VD metabolism and the VD receptor axis in AML, with certain genetic backgrounds and targeted analogs potentially optimizing differentiation and therapeutic responses [64].

A study found that adding vitamins C and D to intensive chemotherapy for AML was safe, was associated with fewer severe adverse events (grade 3–4), helped restore VD levels before transplantation, and correlated with improved overall survival in patients carrying an NPM1 mutation [65].

Separately, research has explored how genetic variations in vitamin D-binding protein (VDBP) influence AML risk and VD status. Specifically, investigators examined the VDBP gene variants rs7041 and rs4588 in AML patients and healthy controls, assessing their relationship with serum 25(OH)D levels [66]. The findings showed that the rs4588 CA and AA genotypes were significantly associated with increased AML susceptibility and a high prevalence of VD deficiency among AML patients [66]. In contrast, under TG-codominant and -dominant genetic models, rs7041 variants appeared to confer some protective effect against AML. Moreover, VD deficiency was more common among AML patients carrying mutant alleles of rs7041 and rs4588. These results suggest that both VD status and interactions with VDBP genetic variants may influence AML risk, highlighting VD deficiency as a potentially important molecular factor in AML risk assessment [66].

In chronic myeloid leukemia (CML), data on VD status are more limited than for other leukemia subtypes. In one study of 84 patients with CML, nearly half were found to have insufficient VD levels, and those with lower 25(OH)D concentrations had higher Sokal scores and were less likely to achieve a major molecular response after 12 months of imatinib therapy [61]. Some reports have also suggested that genetic variants in the VD receptor may influence the response to imatinib, pointing to a possible pharmacogenetic role. However, these observations come from relatively small cohorts and need to be confirmed in larger, prospective studies. The lack of robust evidence specifically addressing VD in CML represents a clear knowledge gap that deserves further attention.

Acute lymphoblastic leukemia (ALL) is the most common pediatric malignancy, with bimodal incidence peaks at 2–5 years of age and after 50 years of age. B-ALL arises from arrested development during B-cell precursor differentiation, whereas T-ALL results from disrupted thymocyte differentiation. Although VDR-dependent differentiation has shown promise in AML, its role in lymphopoiesis and ALL remains poorly understood. Some in vitro studies suggest that 1,25(OH)_2_D_3_ does not affect lymphoblastic leukemia cell proliferation and may impair dexamethasone cytotoxicity, whereas other studies report that 1,25(OH)_2_D_3_ inhibits the growth of normal and malignant lymphoid progenitors despite undetectable VDR expression on leukemic blasts, implying leukemia-extrinsic effects [10].

Given the variability in in vitro conditions, in vivo investigations in humans and animal models are needed to distinguish the intrinsic from extrinsic effects of 1,25(OH)_2_D_3_ on leukemia and lymphoid differentiation. Emerging evidence also indicates that 1,25(OH)_2_D_3_ may influence B-cell differentiation stages, including plasma cell and memory B-cell formation, through interactions with key transcription factors, such as Blimp-1, XBP-1, IRF-4, Pax5, and MITF. Pax5 mutations are common in B-ALL, and Pax5 dysregulation can drive lineage plasticity. Collectively, a system biology approach to 1,25(OH)_2_D_3_ signaling and its crosstalk with transcriptional networks, including Bcl-6, HIF-1α, C/EBPα, STATs, and RUNX2, may help identify targets for manipulating lymphoid differentiation and advancing 1,25(OH)_2_D_3_-based differentiation strategies in leukemia [67].

A study of children with newly diagnosed ALL showed that VD deficiency was present in 39% of patients, with no seasonal variation in VD levels. Patients with optimal 25(OH)D levels tended to have more severe thrombocytopenia and required platelet transfusions more frequently. Higher 25(OH)D levels were also associated with more favorable prognostic indicators, including B-cell phenotype and hyperdiploidy [68].

Calcitriol, the active form of VD, has been shown to influence VDR gene transcription, while sufficient VD availability promotes VDR production in target cells, thereby increasing receptor availability and activity. In leukemia, VD may also help regulate VDR expression and function in immune cells, where VDR contributes to the control of cell growth and inflammation [69].

One study examined the interplay between the OPG/RANK/RANKL axis, VD status, and serum magnesium in ALL survivors. The cohort included 60 ALL survivors treated with chemotherapy and 60 matched controls. VD levels correlated positively with magnesium, calcium, and OPG, and negatively with RANK and RANKL. These findings indicate a high prevalence of VD insufficiency, reduced magnesium levels, and disrupted OPG/RANK/RANKL signaling in ALL survivors, which may contribute to impaired bone remodeling [70].

Higher serum 25(OH)D_3_ levels have been suggested to offer a protective effect against the development of CLL [71]. Among newly diagnosed patients with CLL, approximately 30.5% were 25(OH)D_3_-insufficient, and insufficiency was significantly associated with a shorter time to treatment (TTT) and poorer overall survival (OS) [72]. Calcitriol can inhibit the growth of malignant B-cell progenitors from ALL without altering their viability or differentiation [73].

EB1089, a calcitriol analog, has shown notable antitumor activity in B-cell malignancies. In B-CLL patient cells, EB1089 induced apoptosis through a cell-cycle-independent mechanism, accompanied by decreased Bcl-2 and Mcl-1 expression, reduced ERK activity, and activation of p38 MAPK and caspase-3 [74]. The apoptotic response to EB1089 was selective for malignant B cells, whereas normal B cells from healthy donors showed lower sensitivity [75]. EB1089 also reduced growth and viability in NCI-H929 myeloma cells, promoting G_1_ cell-cycle arrest and the activation of apoptotic pathways, including caspase-3 activation, p38 MAPK activation, PARP degradation, and reduced Bcl-2 expression [75].

In CLL higher serum 25(OH)D_3_ levels have been suggested to exert a protective effect, whereas VD insufficiency has been associated with shorter time to first treatment and poorer overall survival [71,72]. These observations are biologically plausible because CLL cells express VDR, and calcitriol or VD analogues have shown anti-tumor activity in malignant B cells in vitro, including inhibition of malignant B-cell progenitor growth, induction of apoptosis, modulation of Bcl-2/Mcl-1 expression, MAPK pathway activation, and caspase-3–dependent effects [73,74,75]. Clinically, VD replacement has been proposed as a potentially modifiable supportive factor in early-stage CLL; a prospective Mayo Clinic study showed that supplementation can safely increase serum 25(OH)D levels, whereas a large retrospective analysis suggested an association between supplementation and longer time to first treatment [76,77]. However, these data remain insufficient to prove a disease-modifying effect. Therefore, VD insufficiency should currently be regarded as a potentially modifiable supportive-care factor rather than an established therapeutic target in CLL.

The summary of evidence regarding VD status by leukemia subtype is presented in Table 2.

A growing body of evidence across multiple NHL subtypes indicates that low circulating 25(OH)D_3_ levels are common and may adversely affect outcomes. In a cohort of 983 newly diagnosed NHL patients, including patients with DLBCL and T-cell lymphoma, 44% had insufficient 25(OH)D_3_ levels. Deficiency was associated with shorter event-free survival and overall survival in DLBCL and T-cell lymphoma, although this association was not observed across all NHL subtypes [10,78].

VD deficiency has also been associated with poorer survival in follicular lymphoma [79]. In patients with DLBCL receiving rituximab, VD deficiency correlated with shorter event-free survival (EFS), while supplementation enhanced rituximab-mediated cytotoxicity [80].

Patients with cutaneous T-cell lymphoma (CTCL), including Sézary syndrome and mycosis fungoides, similarly show low 25(OH)D_3_ levels, although VD_2_ supplementation did not improve treatment responses [81,82]. VDR expression is high in the majority of B-cell Hodgkin lymphoma samples [83], but low in B-cell NHL overall. Certain VDR single-nucleotide polymorphisms, including rs3819545 and rs2239186, and a CYP24A1 polymorphism, rs2762939, have been linked to NHL and its subtypes [84].

In vitro, calcitriol and the noncalcemic analog MC903 can induce differentiation and suppress proliferation in cell lines representative of follicular NHL, although effective concentrations are often supraphysiological. Nevertheless, clinical responses have been observed at lower doses, possibly through effects on CD4^+^ T cells that support lymphoma proliferation rather than direct effects on tumor cells [85]. In DLBCL cell models, calcitriol and its analogs can trigger necrosis, suggesting that VD-mediated mechanisms may vary according to lymphoma subtype [86].

Across 4502 patients from 14 studies, lower VD status at diagnosis was associated with significantly poorer OS in lymphoid malignancies, with no significant heterogeneity, although a publication bias signal was detected in the OS analysis. The progression-free survival (PFS), analyzed in 3436 patients from 13 studies, was also poorer among patients with low VD status, although notable heterogeneity was observed; no publication bias was detected for PFS [8].

In DLBCL, data from 1272 patients across five studies showed that low-VD status was associated with poorer OS and PFS. In follicular lymphoma (FL), data from 1065 patients across three cohorts showed that low-VD status correlated with worse OS, with no significant heterogeneity [8].

In Hodgkin lymphoma (HL), OS data from 351 patients indicated worse survival among patients with low-VD status. In T-cell lymphoma (TCL), data from 163 patients across two studies for OS and 414 patients across three studies for PFS showed that low-VD status was associated with worse outcomes for both OS and PFS. Subgroup analyses suggested that prognosis may be particularly poor in extranodal NK/T-cell lymphoma [8] (Table 3).

Regarding 25(OH)D status in patients with myeloproliferative neoplasms (MPNs) and myelodysplastic syndromes (MDSs), one study measured 25(OH)D levels in 409 patients, including 247 with primary myelofibrosis (PMF), 74 with de novo MDSs, 63 with polycythemia vera (PV), and 25 with essential thrombocythemia (ET). VD insufficiency was most prevalent in PMF and PV, affecting approximately 48% and 43% of patients, respectively, and was less common in ET and MDS, affecting approximately 28% of patients in each group. Severe deficiency was more common in ET and PMF than in PV and MDS: 12% and 9% versus 3% and 1%, respectively [61].

## 6. Vitamin D’s Effects on Hematopoietic Cells

**Figure 3:**
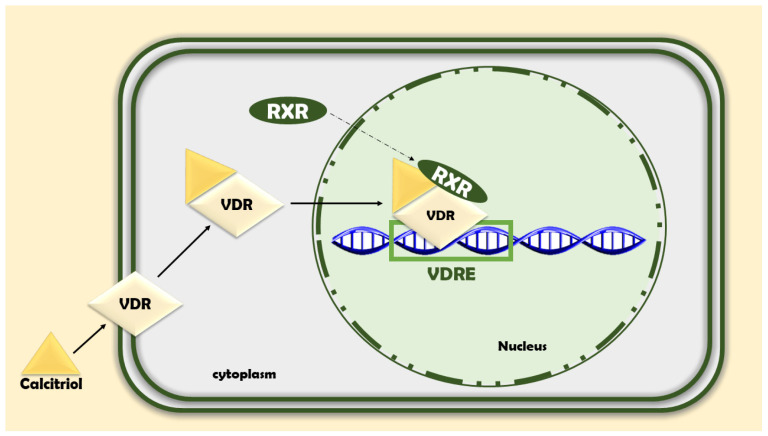
Schematic representation of binding of calcitriol (1,25-dihydroxyvitamin D3) to the vitamin D receptor (VDR) triggers nuclear translocation, where the VDR–RXR complex engages VD response elements (VDREs) to regulate target gene transcription. VDR: vitamin D receptor; RXR: retinoid X receptor; VDRE: vitamin D response element.

As discussed in previous sections, the role of VD in acute and chronic leukemia is largely mediated by the interaction between its active form and VDR, which triggers multiple signaling pathways in hematopoietic cells. Upon ligand binding, 1,25(OH)_2_D_3_ promotes VDR homodimerization or heterodimerization with RXR, allowing the complex to interact with VD response elements (VDREs) and regulate target gene transcription [87,88]. VDR can also interact with retinoic acid receptor (RAR), thereby promoting granulocytic differentiation [89] (Figure 3).

Experimental data suggest competition between RAR/RXR and VDR/RXR dimers for RXR, and the balance between these complexes may influence granulopoiesis and monopoiesis [90]. The VDR–RXR interaction is complex; retinoic acid and VD may mutually enhance each other’s effects, with VDR activity increasing when RAR binds RXR [87]. VDR-knockout mice show normal hematopoiesis under baseline conditions; however, VD derivatives may influence later stages of hematopoietic differentiation [88,91].

VD-triggered signaling converges on Raf-1 and intersects with multiple pathways, including lipid signaling through the protein kinase C (PKC) pathway, the PI3K/AKT pathway, and MAPK signaling [92,93]. Activation of these pathways supports hematopoietic differentiation, with PKC acting as a key mediator [92,93].

1,25(OH)_2_D_3_ influences multiple signaling pathways that drive hematopoietic differentiation. It can activate MAPK pathways and interact with PKC, a key mediator of hematopoietic cell differentiation [94]. In lipid signaling, 1,25(OH)_2_D_3_ increases sphingomyelinase activity in HL-60 cells, thereby raising ceramide levels and enhancing VD-driven differentiation [95,96]. 1,25(OH)_2_D_3_ can also engage the PI3K/AKT signaling axis, contributing to the assembly of a VDR–PI3K complex. This pathway operates in parallel with MAPK signaling and supports cellular differentiation [7].

### 6.1. The Impact of 1,25(OH)_2_D_3_ on MAPK Signaling Pathways

MAPK signaling pathways are central to translating extracellular cues into cellular decisions regarding proliferation, differentiation, and survival in hematopoietic cells. Dysregulated MAPK signaling contributes to myeloid transformation and leukemia, while also mediating 1,25(OH)_2_D_3_-dependent effects on proliferation, differentiation, and apoptosis in AML [97,98].

**Figure 4:**
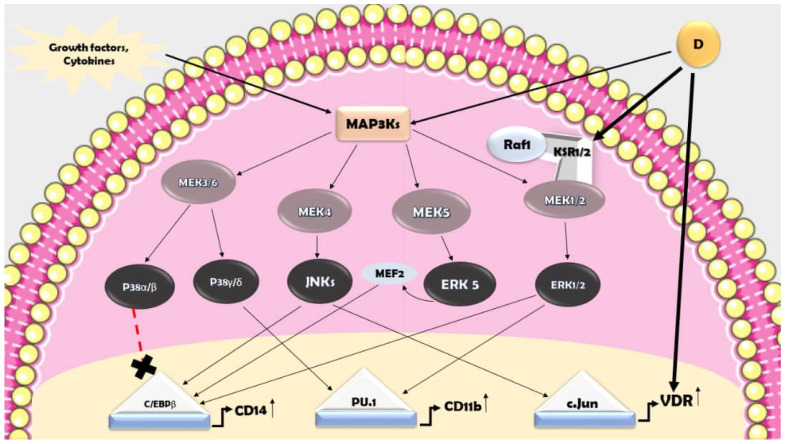
VD signaling interfaces with MAPK pathways to influence transcriptional outcomes. Upon VD exposure, MAPK activity modulates VDR target gene expression through the MEK/ERK axis, linking extracellular signals to genomic responses. Solid arrows represent pathway/activation; the red dashed line with an ‘X’ denotes inhibition; the upward arrow (↑) indicates an increase. MAPK: mitogen-activated protein kinase; Raf: RAF kinase family; KSR: kinase suppressor of Ras; MEK: MAP kinase/ERK; ERK: extracellular signal-regulated kinase; JNK: c-Jun N-terminal kinase.

The MAPK family consists of serine/threonine kinases that participate in four major signaling cascades: (1) extracellular signal-regulated kinase (ERK), (2) p38 kinase, (3) c-Jun N-terminal kinase (JNK), and (4) ERK5 signaling [99] (Figure 4).

VD has also been proposed to promote differentiation by disrupting the AKT–Raf-1 complex, leading to increased Raf-1 levels and activation of the Raf/MEK/ERK MAPK cascade. Overall, three MAPK pathways have been linked to 1,25(OH)_2_D_3_-induced growth arrest and differentiation: Raf-1/MEK/ERK, JNK/MAPK, and p38/MAPK [92].

Activated MAPKs phosphorylate a variety of downstream targets, including transcription factors such as c-Jun, Elk-1, C/EBPs, and MEF2C, as well as apoptosis-regulating proteins such as BIM, which is pro-apoptotic, and MCL-1, which is anti-apoptotic, thereby shaping cell fate toward survival or death [100,101].

In AML, the MEK/ERK axis may be constitutively active in some samples, and upstream signals activating Raf-1/MEK1/2 drive ERK1/2 activation. This RAF/MEK/ERK signaling pathway is associated with poorer prognosis and is considered a potential therapeutic target [102].

The MEK1/ERK1/2 pathway can be activated by a wide range of extracellular signals, including cytokines and growth factors, which sequentially activate Raf-1, MEK1/2, and ERK1/2. Of clinical interest, activation of the RAF/MEK/ERK pathway is associated with poor prognosis and represents a promising target for therapeutic intervention [102,103,104].

Activated ERK translocates into the nucleus and activates several nuclear transcription factors that are important for myeloid differentiation, including Elk-1, MEF2C, and C/EBPs. ERK1/2 may also be activated through the formation of a signaling complex involving multiple kinases and the scaffold protein kinase suppressor of Ras-1 (KSR-1) [105].

The MEK1/2–ERK1/2 cascade is a key regulator of monocytic differentiation in AML cells and may serve as a biomarker of early monocytic differentiation. The p38 and JNK MAPK pathways respond to pro-inflammatory cytokines and cellular stress, and JNK may also be activated by VD metabolites [106,107]. Activation of p38 involves the upstream kinases MKK3/MKK6, which then phosphorylate downstream targets such as MAPK-activated protein kinase 2 [107].

The JNK family, consisting of JNK1, JNK2, and JNK3, functions as a group of stress-activated kinases. These kinases are activated by upstream kinases such as MKK4/MKK7 and regulate gene expression through transcription factors including **c**-Jun, ATF-2, and Elk-1, thereby influencing proliferation, differentiation, and survival [108].

c-Jun is essential for monocytic differentiation in human AML and functions within the AP-1 transcriptional complex. In AML models, JNK activity has been linked to 1,25(OH)_2_D_3_-induced differentiation and phosphorylation of c-Jun, although JNK2 may act as a negative regulator in some VD-resistant AML cells by antagonizing JNK1 signaling [109,110].

The ERK5 pathway, also known as BMK1, is less extensively studied in hematopoiesis and AML, but it also contributes to proliferation, differentiation, and survival, with distinct biological outputs. ERK5 is activated via MEKK2/MEKK3, followed by the nuclear translocation and activation of transcription factors such as MEF2C [111,112].

The inhibition of ERK5 signaling, such as through Cot1 kinase, can enhance 1,25(OH)_2_D_3_-induced differentiation by upregulating differentiation-promoting factors and cell-cycle regulators such as p27Kip1 [113].

ERK5 also modulates C/EBPβ expression through MEF2C, thereby influencing the monocytic differentiation marker CD14 and promoting monocytic differentiation. However, ERK5 may also impede full maturation into functional macrophages by downregulating the macrophage colony-stimulating factor receptor (M-CSFR) in AML cells [111].

Given that tumor-associated macrophages (TAMs) often support tumor progression, enhanced ERK5 activity driven by 1,25(OH)_2_D_3_ may help explain some of the observed benefits of VD in solid tumors, potentially through the modulation of macrophage differentiation or function [114,115].

In the context of lymphopoiesis, studies in VDR-knockout mice show that VDR expression is necessary for normal thymic development and the proper function of invariant natural killer T (iNKT) cells, which exhibit functional defects and lack T-bet expression when VDR is absent [116].

In vitro, VD can suppress NK cell development while directing cells toward myeloid differentiation. However, assessments of thymic CD4^+^ and CD8^+^ T cells and regulatory T cells revealed no major differences between wild-type and VDR-deficient mice [117].

In THP-1 cells, the inhibition of PI3K with LY294002 reduces the 1,25(OH)_2_D_3_-induced expression of monocytic markers such as CD14 and CD11b, indicating that the PI3K pathway contributes to differentiation [118,119].

VDR-knockout mice exhibit extramedullary hematopoiesis due to abnormal bone mineralization. In humans, children with VD deficiency-related rickets may develop hematopoietic abnormalities, including anemia, extramedullary hematopoiesis, thrombocytopenia, myelofibrosis, and myelodysplasia [117]. 1,25(OH)_2_D_3_ also influences embryonic hematopoietic stem and progenitor cell (HSPC) numbers through the VDR-dependent regulation of pro-proliferative signals that appear to function independently of calcium flux [117].

Calcitriol and the noncalcemic analog MC903 stimulate differentiation and suppress proliferation in B-cell lymphoma lines, including SU-DHL4 and SU-BUL5, which model follicular NHL with the t(14;18) translocation. Although the effective concentrations in vitro are higher than physiological levels, clinical responses may occur at lower doses, suggesting that the effects may involve immune cells, such as CD4^+^ T cells, that support lymphoma proliferation rather than direct effects on tumor cells alone [85].

Calcitriol also alters pro-inflammatory cytokine production in peripheral blood mononuclear cells (PBMCs) from patients with aplastic anemia. One study found that IFN-γ, TNF-α, IL-10, IL-17A, and TGF-β1 levels were elevated in PBMCs from patients with aplastic anemia, whereas calcitriol treatment significantly decreased the production of IFN-γ, TNF-α, and IL-17A and increased TGF-β1 production [120].

Calcitriol also inhibited the differentiation of Th17 and Th1 cells while increasing Th2 cells [120]. In addition, calcitriol decreased T-bet mRNA levels in cells from patients with aplastic anemia, supporting a shift away from a Th1 phenotype [121]. Several autoimmune and hematological disorders share a similar cytokine profile characterized by increased IFN-γ, IL-10, IL-6, and IL-17, as observed in immune thrombocytopenia, autoimmune hemolytic anemia, Evans syndrome, and chronic idiopathic neutropenia [122].

EB1089, a calcitriol analog, robustly induces apoptosis in B-CLL cells through a cell-cycle-independent mechanism. This apoptotic process is accompanied by reduced Bcl-2 and Mcl-1 expression, decreased ERK activity, and activation of p38 MAPK and caspase-3. Notably, malignant B-CLL cells from patients show similar responses to EB1089 regardless of treatment status, and malignant cells are more sensitive to EB1089 than normal B cells [74].

Calcitriol-induced apoptosis appears to be relatively selective for malignant cells, while sparing normal cells. EB1089 also reduces growth and viability in NCI-H929 myeloma cells [75]. Growth arrest occurs in the G_1_ phase, whereas apoptosis involves increased caspase-3 activity and activation of p38 MAPK. Associated changes include PARP degradation, the inhibition of ERK/p44 activity, and reduced Bcl-2 levels [75].

Calcitriol analogs with modified side chains, such as PRI-1906 and PRI-1907, promote greater differentiation of AML blast cells than calcitriol alone. CD14 expression increases after treatment with either the analogs or calcitriol. The differentiation response varies according to genetic background: blasts from patients with NPM1 mutations or a normal karyotype respond more strongly, whereas blasts with FLT3 mutations show the weakest differentiation response [88].

VD-induced effects on hematopoietic cells extend beyond the well-documented MAPK pathway and involve multiple signaling networks. In addition to promoting differentiation and exerting antiproliferative effects, VD may activate or inhibit several signaling circuits, including the PI3K/AKT/mTOR, NF-κB, and JAK–STAT pathways [123,124].

### 6.2. The Impact of 1,25(OH)_2_D_3_ on the PI3K/AKT/mTor and NF-κB Axis

**Figure 5:**
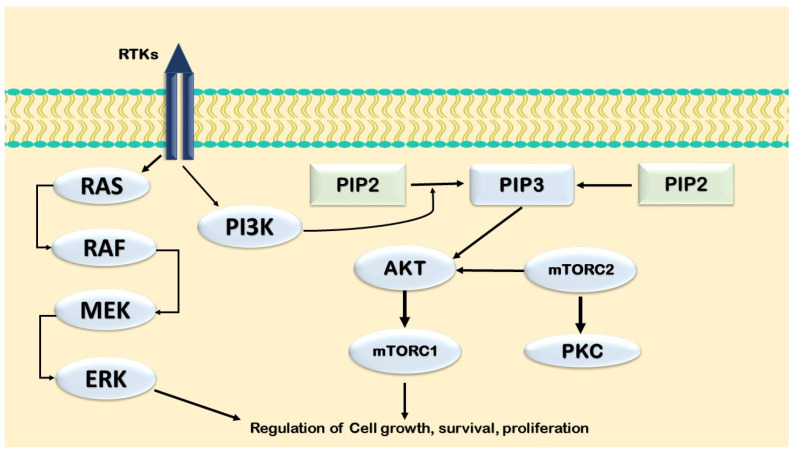
Receptor tyrosine kinase (RTK) activation at the plasma membrane engages PI3K–AKT–mTOR signaling, which converges with the RAS/RAF/ERK cascade to regulate cell growth, survival, and proliferation. MEK: MAP kinase/ERK; ERK: extracellular signal-regulated kinase; PI3K: phosphoinositide 3-kinase; PIP2: phosphatidylinositol-4,5-bisphosphate; PIP3: phosphatidylinositol-3,4,5-trisphosphate; AKT: AKT serine/threonine kinase; mTORC: mechanistic target of rapamycin complex; PKC: protein kinase C.

The PI3K/AKT/mTOR axis is a central regulator of cell growth and survival and is frequently dysregulated in cancers, including AML. Early studies showed that 1,25(OH)_2_D_3_ activates AKT, contributing to anti-apoptotic effects and cell-cycle control in differentiating HL-60 cells. AKT may also influence 1,25(OH)_2_D_3_-induced differentiation through the RAF/MEK/ERK pathway [125] (Figure 5).

In AML, mTOR inhibitors can enhance 1,25(OH)_2_D_3_-driven differentiation, and VD-related analogs may modulate PI3K/AKT/mTOR-dependent immunosuppressive effects, including PI3Kα-mediated increases in steroid sulfatase activity. Beyond hematopoietic cells, AKT/mTOR signaling may broadly transmit VD-mediated signals, suggesting that mTOR may act as a general integrator of VD’s immunomodulatory and antiproliferative effects.

Crosstalk with NF-κB, observed in HL-60 cells, may contribute to VD-mediated immune regulation, with NF-κB suppression enhancing 1,25(OH)_2_D_3_-induced monocytic differentiation. Coordinated activation of the JAK–STAT, p38 MAPK, and NF-κB pathways supports the regulation of 1α-hydroxylase in monocytes, potentially via C/EBPβ. VD also modulates the IL-12/IFN-γ axis through JAK–STAT signaling to promote Th1 responses in some models and may dampen TNF-induced tissue factor expression by inhibiting AP-1 and NF-κB signaling.

Overall, substantial signaling crosstalk underlies hematopoietic responses to VD. In myeloid leukemia, 1,25(OH)_2_D_3_ promotes monocytic differentiation and modulates immune function, providing a mechanistic basis for strategies aimed at inducing differentiation or apoptosis in AML and related leukemias [126,127,128,129].

### 6.3. The Impact of 1,25(OH)_2_D_3_ on the Jak–STAT Pathway

VD may act as a modulator of JAK–STAT signaling, as it has been shown to reduce STAT3 activation in mouse models of experimental diabetes [130] and experimental allergic encephalomyelitis (EAE) [131], as well as in human esophageal squamous cell carcinoma (SCC) cell lines [132].

Inhibiting the JAK–STAT signaling pathway can induce apoptosis in several cancer cell types, including large granular lymphocytic leukemia (LGLL) cells [53]. Therefore, potential pharmacological inhibitors of JAK–STAT signaling are under investigation. However, the limited potency of some STAT inhibitors and their adverse effects currently restrict their clinical application [133].

Furthermore, VD decreases STAT1 activation and pro-inflammatory cytokine production in a mouse model of EAE, which correlates with reduced disease severity and improved clinical symptoms [131]. In hepatocellular carcinoma, this reduction in pro-inflammatory cytokines has been hypothesized to occur in a p27^Kip1-dependent manner [134].

Recently, the effects of VD on the JAK–STAT pathway in LGLL have been investigated. Although 30–40% of LGLL patients harbor somatic activating mutations in STAT3 [120,122], constitutive activation of STAT3 and STAT1 is also observed in patients without STAT3 mutations [53]. To date, no activating mutations in STAT1 have been identified [133].

**Figure 6:**
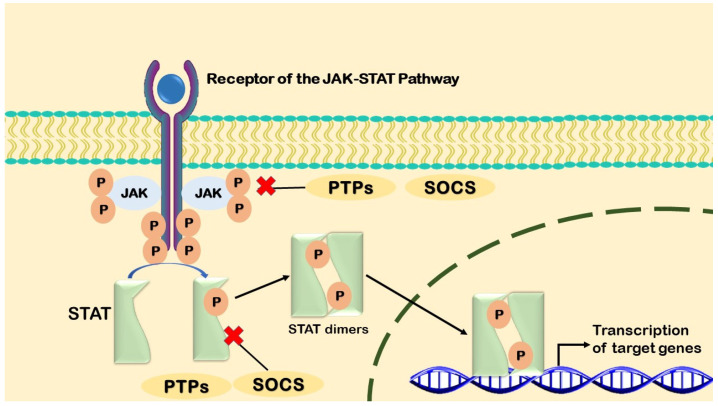
A schematic representation of the JAK–STAT pathway. The activation of JAK kinases downstream of cytokine receptors drives STAT phosphorylation and nuclear translocation. Protein tyrosine phosphatases (PTPs) attenuate signaling by dephosphorylating JAKs/STATs, while SOCS proteins impose negative feedback, collectively shaping the magnitude and duration of STAT target gene expression. The red ‘X’ indicates inhibition. JAK: Janus kinase; STAT: signal transducer and activator of transcription; PTP: protein tyrosine phosphatase; SOCS: suppressor of cytokine signaling.

The increased activation of JAK–STAT signaling in patients without STAT3 mutations may result from aberrant cytokine signaling, particularly involving IL-6, and the repression of endogenous JAK–STAT inhibitors, especially suppressor of cytokine signaling 3 (SOCS3) [130] (Figure 6).

This SOCS suppression and increased IL-6 production have also been observed in other cancers [131,132,134]. Recent findings indicate that calcitriol increases VDR protein levels, reduces STAT1 and STAT3 activation, and decreases pro-inflammatory cytokine production, particularly IFN-γ production [133]. Cell viability remained unchanged in both the T-LGLL cell line and primary patient cells, supporting a potential shift from a pro-inflammatory phenotype toward a more anti-inflammatory state [133].

Overall, VD may exert antiproliferative and pro-differentiation effects through the VDR-mediated transcriptional regulation and modulation of key oncogenic signaling pathways (Table 4).

## 7. Vitamin D Supplementation in the Management of Leukemia and Chemotherapy: Opportunities and Challenges

Among other considerations, hypovitaminosis D occupies an important place, and the possibility of VD supplementation serving as an adjunct therapy in leukemia management, in addition to conventional therapy, represents a promising approach. This is further supported by the development of synthetic VD analogs designed to improve antitumor efficacy. The well-established effects of VD on cellular proliferation, differentiation, apoptosis, and immune modulation have attracted considerable attention to VD supplementation as part of a new strategy to address different cancers, including leukemia. VD deficiency is a consistent finding in leukemia patients, with more severe hypovitaminosis often observed in those undergoing chemotherapy. The prevention and treatment of VD deficiency in these patients should therefore be considered. Hypovitaminosis D is common among leukemia patients, especially those receiving intensive chemotherapy, in whom limited sun exposure, altered metabolism, and nutritional deficits may aggravate the deficiency [78,135,136]. These considerations provide a rationale for the further discussion of VD-based therapy as an add-on to standard treatment, particularly through the use of VD analogs with a reduced risk of hypercalcemia and potentially enhanced antitumor activity [136].

### 7.1. Vitamin D Supplementation in Leukemia Patients

A substantial number of studies have suggested that adequate plasma VD levels in patients with hematologic malignancies may be associated with improved clinical outcomes. Shanafelt et al. [72] found that CLL patients with low serum VD levels had significantly shorter progression-free survival and overall survival than those with sufficient levels, emphasizing the prognostic importance of VD status. Similar observations have also been reported in AML cohorts, where VD deficiency has been correlated with adverse cytogenetic features and a higher rate of disease relapse [10,137]. These findings support the correction of VD deficiency as part of supportive care, but they do not establish supplementation as an anti-leukemic intervention [10].

There are currently no leukemia-specific guidelines recommending VD supplementation as part of anti-leukemic therapy [78]. However, correction of VD deficiency is well justified as a component of comprehensive supportive care for patients with hematologic malignancies [135]. Given the high prevalence of VD deficiency in leukemia and the probable influence of treatment-related factors on VD metabolism, routine serum 25(OH)D testing at diagnosis or before the initiation of intensive treatment appears reasonable [136]. Supplementation should primarily aim to correct deficiency and protect skeletal health, while avoiding excessively high doses. Clinical management decisions should be individualized according to baseline VD status, kidney function, serum calcium levels, concomitant medications, and ongoing anticancer treatment [72].

In addition, patients with renal impairment, prolonged immobilization, or conditions predisposing to hypercalcemia require special caution [137]. In such cases, regular monitoring of serum calcium, renal function, and 25(OH)D levels is recommended during high-dose supplementation [10,138]. Existing recommendations for leukemia patients are briefly summarized in Table 5.

### 7.2. Impact on Chemotherapy Tolerance and Efficacy

VD has been proposed to support immune regulation and cellular homeostasis, which may contribute to better tolerance of cytotoxic therapies. In animal experiments, 1,25(OH)_2_D_3_ enhanced the anticancer effects of cytarabine by promoting differentiation and increasing the susceptibility of AML cells to cytarabine-induced cytotoxicity [138]. It was reported that high concentrations of VD analogs could enhance chemotherapy-induced leukemic cell death, supporting a potential combined-treatment strategy [139]. Moreover, supplementation may help reduce selected chemotherapy-related toxicities, such as mucositis and fatigue, possibly through anti-inflammatory mechanisms [140]. However, the optimal dosing strategy, particularly in severely immunocompromised patients, remains unknown and should be determined in clinical trials [140,141].

Despite supportive observational data and promising preclinical findings, strong clinical evidence for VD supplementation as an adjuvant therapeutic strategy in leukemia is still lacking [8]. Most available studies are retrospective, observational, or based on relatively small and heterogeneous patient cohorts, making it difficult to determine causality or the magnitude of clinical benefit [65]. Another important limitation is the substantial variability in baseline VD status, supplementation regimens, dosing strategies, treatment duration, leukemia subtypes, and concurrent therapies, which limits comparability across studies [77]. To date, sufficiently large randomized controlled trials with clinically meaningful endpoints, such as overall survival, relapse-free survival, treatment response, infection rates, and quality of life, have not been conducted [78,135]. Therefore, although the correction of VD deficiency is appropriate as part of standard supportive care for skeletal and general health maintenance, current evidence does not support VD supplementation as a disease-modifying therapeutic intervention in leukemia [72,136]. Future multicenter randomized controlled trials with standardized supplementation protocols and stratification according to baseline VD status and leukemia subtype are essential to determine whether VD supplementation provides direct clinical benefits beyond correction of deficiency [10,137].

### 7.3. Development of Vitamin D Analogs

The therapeutic use of calcitriol in cancer has traditionally been limited by the risk of hypercalcemia at pharmacologically active doses [142]. To overcome this limitation, several VD analogs, including EB1089, calcipotriol, and paricalcitol, have been developed. These compounds retain antiproliferative activity similar to that of the parent compound, while showing a substantially reduced calcemic potential [142,143,144,145]. Swami et al. [146] reported that EB1089 inhibited AML cell proliferation while maintaining safer calcium homeostasis, supporting the rationale for analog-based therapeutic approaches. In addition, some VD analogs may exhibit improved interaction with VDR, leading to the more effective modulation of gene expression profiles and, potentially, stronger anti-leukemic activity [144].

### 7.4. Vitamin D-Mediated Signaling and Apoptotic Pathways in Leukemia

At the molecular level, VD and its active metabolites affect leukemic cells through multiple genomic and non-genomic signaling pathways. VDR is the main mediator of the genomic effects of VD. As a ligand-activated transcription factor, VDR forms a heterodimer with RXR and, after ligand binding, translocates to the nucleus, where it binds to VD response elements (VDREs) within target genes and regulates transcriptional networks controlling cell proliferation, differentiation, and apoptosis [138,142,144]. In leukemic blasts, VDR activation promotes the upregulation of CDKN1A (p21) and CDKN1B (p27), which is associated with G_0_/G_1_ cell-cycle arrest and the inhibition of cyclin-dependent kinase activity [147].

In addition to genomic pathways, VD also influences several non-genomic signaling cascades, particularly the MAPK, PI3K/AKT, and NF-κB pathways. Activation of p38 MAPK and ERK1/2 has been shown to be central to VD-induced monocytic differentiation in AML cell lines. Moreover, apoptotic sensitivity is further increased when PI3K/AKT signaling is simultaneously inhibited [139,148]. Notably, 1,25(OH)_2_D_3_ suppresses constitutive NF-κB activity, which is implicated in several leukemia subtypes, thereby reducing the expression of anti-apoptotic genes such as BCL-2, BCL-XL, and MCL1 [140,143]. This suppression may increase leukemic cells’ susceptibility to apoptosis induced by chemotherapy and cytokine-mediated signaling. Mechanistically, VD may activate the intrinsic, mitochondrial apoptotic pathway by modulating the BCL-2 family and triggering the caspase cascade. The upregulation of BAX and BAK, together with the downregulation of BCL2, promotes mitochondrial outer membrane permeabilization, cytochrome c release, and the subsequent activation of caspase-9 and caspase-3 [146,149,150]. Moreover, VD derivatives have been reported to enhance Fas/FasL-mediated extrinsic apoptosis, particularly in acute promyelocytic leukemia (APL) cells, by increasing the expression of FAS and its ligand, thereby strengthening death receptor signaling [10].

In addition, evidence implicates STAT3 and β-catenin signaling in the crosstalk between VD pathways and leukemogenesis. Calcitriol reduces phosphorylated STAT3 levels, resulting in decreased transcription of proliferative genes such as MYC and CCND1 [151]. Furthermore, the VDR-mediated inhibition of β-catenin nuclear translocation may suppress Wnt-driven self-renewal, a process involved in leukemic stem cell persistence [148,152].

### 7.5. Clinical Perspectives and Limitations

Despite strong biological plausibility and encouraging preclinical findings, the clinical role of VD supplementation in leukemia remains incompletely defined [147,148,152]. Available studies differ substantially in leukemia subtype, treatment phase, baseline VD status, supplementation dose, patient population, and outcome measures, which limits direct comparison across studies and makes it difficult to define a universal supplementation strategy [147,148]. Potential drug–nutrient interactions, particularly with agents metabolized through cytochrome P450 enzymes, should also be considered when supplementation is used during active leukemia treatment [152]. At present, correction of deficiency is justified for general health, skeletal protection, immune support, and supportive-care purposes, but not as a stand-alone anti-leukemic treatment [147,148,152]. Future studies should focus on biomarker-guided supplementation, safety monitoring, and clinically meaningful endpoints in well-defined leukemia populations [147,148].

## 8. Discussion

Experimental studies support a biologically plausible role for VD signaling in leukemic cell differentiation, apoptosis, autophagy, inflammatory regulation, and immune function [124,125,126,127,128]. However, clinical interpretation requires caution. Most available human data are observational and may be influenced by reverse causality and residual confounding, including disease severity, nutritional status, systemic inflammation, reduced sunlight exposure, hepatic or renal dysfunction, obesity, and treatment-related factors [8,51,123]. Therefore, low 25(OH)D levels may represent both a contributor to impaired host defense and a marker of poorer clinical condition [51,123]. This distinction is important when interpreting associations between VD status and leukemia outcomes [8,51].

One of the main challenges in interpreting the available literature is the considerable heterogeneity in study design, patient populations, and sample sizes. To provide a clearer framework for readers, we stratified the evidence cited in this review according to the study design and sample size. We defined three broad evidence tiers: meta-analyses and large prospective cohort studies (*n* > 500) were considered to provide the strongest evidence; moderate-sized cohort studies (*n* = 100–500) were considered intermediate evidence; and smaller case series, retrospective analyses, and pilot studies (*n* < 100) were considered the most limited in terms of generalizability. This stratification is not intended to replace critical appraisal of individual studies, but rather to help readers assess the relative weight of the findings discussed throughout this review (Table 6).

Mechanistically, VD, through both genomic and non-genomic signaling pathways, primarily via VDR-mediated transcriptional regulation, can regulate cell proliferation, differentiation, and cell death. After 1,25(OH)_2_D_3_ binds to the VDR–RXR complex, it modulates the expression of key regulatory genes, including p21, p27, and C/EBPβ, resulting in the maturation of leukemic blasts and G_1_-phase cell-cycle arrest [140,143,148]. At the same time, intracellular signaling pathways, including MAPK, PI3K/AKT/mTOR, and NF-κB, may be activated or inhibited, thereby influencing the balance between differentiation, survival, and cell death [97,125,149]. Crosstalk between these pathways and the JAK–STAT and β-catenin signaling axes may contribute to the anti-leukemogenic effects of VD by repressing oncogenic transcription factors such as MYC, CCND1, and STAT3 [130,147,148]. In this context, the potential role of VD in limiting leukemic progression may be partly related to its ability to inhibit β-catenin nuclear translocation, thereby reducing the survival capacity and therapy resistance of leukemic stem cells [148,152].

Although substantial experimental evidence supports a biological role for VD signaling in the regulation of leukemic cell proliferation, differentiation, apoptosis, and immune responses, considerable caution is required when interpreting associations between clinical markers of VD deficiency and adverse leukemia outcomes. Most available clinical evidence is derived from observational studies and is therefore susceptible to reverse causality and residual confounding by disease stage and severity. Patients with leukemia frequently experience reduced dietary intake, malnutrition, systemic inflammation, impaired hepatic or renal conversion of VD into its active metabolites, prolonged hospitalization, reduced physical activity, and limited sunlight exposure, all of which may contribute to lower circulating 25(OH)D levels [8,51,123].

Therefore, VD deficiency may partly reflect disease severity rather than act as an independent causal determinant of leukemia progression. Nevertheless, a contributory biological role of impaired VD signaling cannot be excluded. Experimental studies have shown that VDR activation modulates several pathways relevant to leukemogenesis, including MAPK, PI3K/AKT/mTOR, JAK/STAT, NF-κB, and β-catenin signaling. These pathways are involved in the regulation of proliferation, differentiation, apoptosis, immune function, oxidative stress, mitochondrial metabolism, epigenetic regulation, and bone marrow microenvironmental interactions [87,92,126,130].

Taken together, the relationship between VD deficiency and leukemia outcomes is likely complex and potentially bidirectional. Leukemia and its treatment may contribute to reduced VD levels, whereas impaired VD signaling may, according to available experimental and observational evidence, influence disease progression, immune dysregulation, and treatment responsiveness. However, current data are insufficient to establish a direct causal relationship.

Accordingly, VD status should not be interpreted as a stand-alone prognostic marker, but rather within a broader network biology framework that integrates molecular, metabolic, immunological, nutritional, and clinical factors. Future large-scale prospective studies, Mendelian randomization analyses, and adequately powered randomized controlled trials are needed to distinguish causality from association, identify patient subgroups most likely to benefit from VD correction, and determine whether VD supplementation provides clinically meaningful benefits beyond standard supportive care [10,72,78,135,136,137].

In AML, one potential mechanism through which VD may exert therapeutic effects is the induction of leukemic cell differentiation. This is supported by evidence implicating autophagy-related pathways, including Beclin-1 activation and mTOR suppression, which may represent an attractive mechanism for combination with current standard-of-care therapies [55]. In CLL, VD deficiency has been associated with markedly shorter time to treatment and overall survival, while observational studies suggest that the correction of deficiency may be associated with improved outcomes [76,77]. Thus, although these findings remain limited, they indicate a potential prognostic and supportive-care opportunity for addressing VD deficiency in leukemic populations.

However, translating molecular promise into clinical practice remains challenging. Therapeutic implementation is complicated by the variability in baseline VD status, polymorphisms in VDR and CYP24A1, and the risk of hypercalcemia at pharmacological doses [9,29,63]. VD analogs such as EB1089, paricalcitol, and calcipotriol may retain anti-leukemic activity while reducing calcemic adverse effects; however, confirmation in large randomized clinical trials is still lacking [143,144,145,146]. Heterogeneous study designs, small sample sizes, and inconsistent dosing protocols further limit the ability to define clear supplementation strategies.

From a clinical perspective, the routine correction of VD deficiency in patients with leukemia appears reasonable because deficiency is common and may influence immune function, chemotherapy tolerance, and infection susceptibility [10,72,78,135,136,137,138,139,140]. Nevertheless, VD supplementation should not be considered a stand-alone anti-leukemic treatment, but rather a biologically guided supportive-care strategy that complements conventional therapy. The integration of genomic profiling, including VDR and CYP polymorphisms, with real-time monitoring of VD status may help individualize supplementation and reduce the risk of over-supplementation.

Overall, the available evidence suggests that VD is a relevant modulator of leukemic biology and a potential component of supportive care. Achieving adequate VD status may improve treatment tolerance, support immune function, reduce complications, and potentially contribute to better long-term outcomes, although this requires confirmation in well-designed prospective clinical trials. Therefore, the next step in integrating VD into hematologic oncology should not be empirical dosing, but precision supplementation guided by biochemical monitoring, molecular profiling, and clinical context.

## 9. Conclusions

In conclusion, VD deficiency appears to be a frequent and clinically relevant finding in patients with leukemia, particularly among those receiving intensive chemotherapy. Current evidence suggests that VD may influence leukemic biology through VDR-mediated genomic and non-genomic mechanisms, including the regulation of hematopoietic differentiation, cell-cycle progression, apoptosis, autophagy, inflammatory signaling, and immune responses. These effects are mediated, at least in part, through the MAPK, PI3K/AKT/mTOR, NF-κB, JAK–STAT, and β-catenin pathways.

Clinical studies suggest that low 25(OH)D levels are associated with poorer outcomes in selected leukemia subtypes, including reduced treatment response, shorter overall or relapse-free survival, and shorter time to treatment. However, these findings should be interpreted as associative rather than causal. VD supplementation should therefore not be presented as an independent anti-leukemic therapy. Instead, assessment and correction of deficiency may represent a rational, low-toxicity component of supportive care, particularly in patients at risk of hypovitaminosis D during intensive therapy.

Future research should define which patients are most likely to benefit from supplementation and determine whether individualized strategies based on baseline 25(OH)D status, leukemia subtype, treatment phase, VDR/CYP polymorphisms, and safety monitoring can improve clinically meaningful outcomes.

## Figures and Tables

**Table 1:** Summary of studies evaluating VD status in hematologic malignancies.

Study	Population	VD Assessment (25(OH)D)	Assay Method	VDR/RXR Analysis	Key Findings	Standardization (NIST/LC-MS/MS)
[49]	137 hematologic malignancies + 60 controls	Serum 25(OH)D	ELISA	RT-PCR (VDR, RXR mRNA)	Significant correlation between VD levels and disease status (*p* < 0.001); age associated with risk	Not reported
[50]	105 leukemia patients	Serum 25(OH)D_3_	Immunoassay (reported serum measurement)	Not assessed	~72% VD-deficient/-insufficient; lower VD linked to higher disease burden	Not reported
[51]	Meta-analysis (7 studies, 2643 patients)	25(OH)D levels	Mixed (various assays across studies)	Not assessed	Low VD associated with shorter OS and RFS	Not standardized across studies
[8]	Hematologic malignancies + lymphoma subtypes	Serum 25(OH)D	Mixed assays across included studies	Not assessed	Low VD at diagnosis associated with poor prognosis; transplant outcomes affected	Not consistently reported

VD, vitamin D; 25(OH)D, 25-hydroxyvitamin D; 25(OH)D_3_, 25-hydroxyvitamin D_3_; VDR, vitamin D receptor; RXR, retinoid X receptor; mRNA, messenger ribonucleic acid; ELISA, enzyme-linked immunosorbent assay; RT-PCR, reverse transcription polymerase chain reaction; OS, overall survival; RFS, relapse-free survival; NIST, National Institute of Standards and Technology; LC-MS/MS, liquid chromatography–tandem mass spectrometry.

**Table 2:** Summary of evidence regarding VD status by leukemia subtype.

Subtype	Number of Studies and Total Patients	Key Findings	Evidence Grade	References
AML	7 studies, 1284 patients	Low VD associated with worse RFS and OS	Moderate	[8,59,65]
ALL	4 studies, 512 patients	Low VD associated with higher relapse risk in pediatric cohorts	Low	[8,68]
CML	2 studies, 136 patients	Low VD associated with poorer molecular response	Low	[61]
CLL	5 studies, 1204 patients	Low VD associated with shorter time to first treatment	Moderate	[8,72,77]
MDS/MPN	3 studies, 409 patients	Low VD common, but outcome association unclear	Low	[61]

VD, vitamin D; OS, overall survival; RFS, relapse-free survival; AML, acute myeloid leukemia; ALL, acute lymphoblastic leukemia; CML, chronic myeloid leukemia; CLL, chronic lymphocytic leukemia; MDS, myelodysplastic syndrome; MPN, myeloproliferative neoplasm. Note: Evidence grade represents an author-developed qualitative framework for summarizing the relative strength of available evidence across leukemia subtypes. It should not be interpreted as a formal GRADE-based assessment and does not replace systematic evaluation of certainty of evidence, risk of bias, or recommendation strength.

**Table 3:** Overview of the reported VD levels in patients with various lymphoma subtypes, including sample size and key findings.

Lymphoma Subtype	Sample Size	Main Findings	Effect Estimates (HR, 95% CI, *p*-Value)
Non-Hodgkin lymphoma (overall)	4502 patients (14 studies)	Low VD linked to poorer OS and PFS in pooled analysis of lymphoid cancers	OS: HR = 2.07 (95% CI: 1.79–2.40); PFS: HR = 1.91 (95% CI: 1.61–2.25)
Diffuse large B-cell lymphoma (DLBCL)	1272 patients (5 studies)	Lower OS and PFS; supplementation may enhance rituximab efficacy	OS: HR = 1.99 (95% CI: 1.27–3.13, *p* = 0.003)
Follicular lymphoma (FL)	1065 patients (3 cohorts)	Low VD correlated with worse OS (significant in one large cohort; not significant in another)	OS: HR = 1.91 (95% CI: 1.45–2.51, *p* < 0.001)
T-cell lymphoma (TCL)	163 patients (2 studies) and 414 patients (3 studies)	Low VD associated with shorter OS and PFS; poorer prognosis in extranodal NK/T-cell lymphoma	OS: HR = 2.01 (95% CI: 1.42–2.85); PFS: HR = 1.73 (95% CI: 1.31–2.28)
Hodgkin lymphoma (HL)	351 patients	Low VD associated with worse OS	OS: HR = 1.58 (95% CI: 1.07–2.34, *p* = 0.02)

VD, vitamin D; OS, overall survival; PFS, progression-free survival; HR, hazard ratio; CI, confidence interval; DLBCL, diffuse large B-cell lymphoma; FL, follicular lymphoma; TCL, T-cell lymphoma; HL, Hodgkin lymphoma; NK/T-cell lymphoma, natural killer/T-cell lymphoma.

**Table 4:** The summary of VD’s main signaling pathways, and their main effects and key targets.

Pathway	Main Effect	Key Targets/Results
VDR/RXR genomic	Cell-cycle arrest, differentiation	p21, p27 ↑ c-MYC ↓ Cyclin-D1↓
MAPK/ERK	Differentiation modulation	AP-1 regulation
NF-κB	Anti-inflammatory, pro-apoptotic	IL-6 ↓ BCL-XL↓
PI3K/AKT/mTOR	Growth inhibition	AKT ↓ mTOR ↓ PTEN ↑
JAK/STAT	Reduced survival signaling	p-STAT3 ↓ c-MYC ↓

↑, increase or upregulation; ↓, decrease or downregulation; VDR, vitamin D receptor; RXR, retinoid X receptor; MAPK, mitogen-activated protein kinase; ERK, extracellular signal-regulated kinase; NF-κB, nuclear factor kappa B; PI3K, phosphoinositide 3-kinase; AKT, protein kinase B; mTOR, mechanistic target of rapamycin; AP-1, activator protein 1; IL-6, interleukin 6; BCL-XL, B-cell lymphoma-extra-large; PTEN, phosphatase and tensin homolog; JAK, Janus kinase; STAT, signal transducer and activator of transcription; p-STAT3, phosphorylated signal transducer and activator of transcription 3; c-MYC, cellular MYC proto-oncogene.

**Table 5:** Practical considerations for VD assessment and supplementation in patients with leukemia based on current clinical practice guidelines and expert recommendations. Recommendations are adapted from clinical practice guidelines and consensus statements on VD supplementation in adult populations and cancer patients [10,72,78,135,136,137,138,139].

Clinical Aspect	Practical Recommendation
Baseline assessment	Measure serum 25(OH)D before or early during treatment, particularly in patients receiving intensive chemotherapy or hematopoietic stem cell transplantation [10,72,78,135,136,137].
Target 25(OH)D level	Maintain serum 25(OH)D ≥30 ng/mL (75 nmol/L); avoid persistent concentrations >50–60 ng/mL unless clinically indicated [138].
Patients requiring supplementation	VD deficiency (<20 ng/mL) or insufficiency (20–29 ng/mL), especially in patients with limited sun exposure, malnutrition, corticosteroid therapy, or prolonged hospitalization [78,135,136].
Suggested maintenance dose	Usually 800–2000 IU/day, individualized according to baseline deficiency and patient characteristics [138].
Severe deficiency	Higher replacement doses may be required according to established endocrine guidelines, followed by maintenance therapy [138,139].
Monitoring	Reassess serum 25(OH)D and calcium approximately 8–12 weeks after initiating supplementation; thereafter according to clinical status [138,139].
Safety considerations	Monitor for hypercalcemia, particularly in patients with renal impairment, immobilization, granulomatous disorders, or concomitant calcium supplementation [10,72,78,135,136,137,138].
Drug interactions	Consider renal function, corticosteroid exposure, anticonvulsants, antifungals, and other medications affecting VD metabolism during chemotherapy [10,72,78,135,136,137].
Current evidence	VD supplementation is recommended for correction of deficiency and bone health; evidence supporting improvement in leukemia-specific survival or treatment response remains insufficient [8,10,72,78,135,136,137].

VD, vitamin D; 25(OH)D, 25-hydroxyvitamin D.

**Table 6:** Stratification of evidence by study type, design, and sample size.

Evidence Type	Study Design and Sample Size	Key Findings	Limitations	Strength of Evidence
Mechanistic (in vitro/animal)	Laboratory studies, animal models	1,25D induces differentiation and apoptosis via VDR, MAPK, PI3K/AKT; regulates autophagy via Beclin1; modulates immune cytokines [62]	Supraphysiological doses; cell line-specific effects; lack of in vivo relevance	Moderate
Observational (clinical)	Cohort studies and meta-analyses and large cohorts (*n* > 500)	Low 25(OH)D associated with worse OS, RFS, TTFT in AML, CLL, DLBCL [8,51,77]	Residual confounding; assay heterogeneity	Moderate–strong
	Cohort studies (*n* = 100–500)	VDR/CYP24A1 polymorphisms associated with outcome [59,65,72,78]	Reverse causation; small sample sizes in some studies	Low–moderate
	Case–control, retrospective (*n* < 100)	VD deficiency common; association with outcomes less consistent [49,50,63,66,68]	High risk of bias; limited generalizability	Low
Interventional (RCTs/pilot)	Small pilot studies, phase I/II trials	Supplementation safe and restores VD levels; some evidence for improved outcomes in NPM1-mutated AML [65]	Very few RCTs; small sample sizes; short follow-up; dosing heterogeneity	Very low

VD, vitamin D; VDR, vitamin D receptor; AML, acute myeloid leukemia; CLL, chronic lymphocytic leukemia; OS, overall survival; RFS, relapse-free survival; TTFT, time to first treatment; RCTs, randomized controlled trials; Note: Evidence grading in this table represents an author-developed qualitative evidence-stratification approach intended to summarize the relative strength of available data. It should not be interpreted as a formal evidence-grading framework, such as GRADE, and does not replace systematic assessment of certainty of evidence, risk of bias, or strength of recommendations.

## Data Availability

We used the PubMed, SCOPUS, and ScienceDirect databases to screen articles for this review. We did not report any data.

## Abbreviations

1,25(OH)_2_D_3_	1,25-dihydroxyvitamin D_3_
15-PGDH	15-hydroxyprostaglandin dehydrogenase
25(OH)D	25-hydroxyvitamin D
25(OH)D_3_	25-hydroxyvitamin D_3_
ALL	acute lymphoblastic leukemia
AML	acute myeloid leukemia
B-ALL	B-cell acute lymphoblastic leukemia
BMD	bone mineral density
C/EBPs	CCAAT/enhancer-binding proteins
CI	confidence interval
CLL	chronic lymphocytic leukemia
CML	chronic myeloid leukemia
COX	Cyclooxygenase
CR	complete remission
CRC	colorectal cancer
cAMP	cyclic adenosine monophosphate
DLBCL	diffuse large B-cell lymphoma
EFS	event-free survival
eGFR	estimated glomerular filtration rate
ELISA	enzyme-linked immunosorbent assay
ERK	extracellular signal-regulated kinase
FL	follicular lymphoma
FLT3	FMS-like tyrosine kinase 3
GRADE	Grading of Recommendations Assessment, Development and Evaluation
Hh	Hedgehog signaling pathway
HL	Hodgkin lymphoma
HR	hazard ratio
IFN	interferon gamma
iNKT	invariant natural killer T cells
JNKs	c-Jun N-terminal kinases
KSR	kinase suppressor of Ras
LC-MS/MS	liquid chromatography–tandem mass spectrometry
MAPK	mitogen-activated protein kinase
MDS	myelodysplastic syndromes
MEF2	myogenic enhancer factor 2
miR-214	microRNA-214
MPN	myeloproliferative neoplasm
mTOR	mammalian target of rapamycin
mRNA	messenger ribonucleic acid
MTX	Methotrexate
NF-κB	nuclear factor kappa B
**NHL**	non-Hodgkin lymphoma
NIST	National Institute of Standards and Technology
non-RTKs	non-receptor tyrosine kinases
nVDR	nuclear vitamin D receptor
OS	overall survival
PGE_2_	prostaglandin E_2_
PI3K	phosphatidylinositol-3 kinase
PKC	protein kinase C
PLA_2_	phospholipase A_2_
PFS	progression-free survival
RAR	retinoic acid receptor
RCTs	randomized controlled trials
RFS	relapse-free survival
RT-PCR	reverse transcription polymerase chain reaction
RXR	retinoid X receptor
SNP	single-nucleotide polymorphism
T-ALL	T-cell acute lymphoblastic leukemia
TCL	T-cell lymphoma
TF	transcription factor
TNF	tumor necrosis factor
TTFT	time to first treatment
UVB	ultraviolet B radiation
VD	vitamin D
VDBP	vitamin D-binding protein
VDR	vitamin D receptor
VDRE	vitamin D response element
